# Topics of the Month

**Published:** 1892-08

**Authors:** 


### TOPICS OF THE MONTH.

At a meeting of the Board of Trustees of the Pan-American Medical Congress, held at the Russell House, Detroit, June 8, 1892, Dr. Henry D. Holton, of Brattleboro, Vt., was elected Chairman; Dr. A. Walter Suiter, Secretary of the Board, also Assistant Secretary-General of the Pan-American Medical Congress; and Dr. A. M. Owen, Treasurer of the Congress, was elected Treasurer of the Board of Trustees. Dr. L. S. McMurtry, of Louisville, Dr. Henry D. Holton, of Brattleboro, and Dr. W. W. Potter, of Buffalo, were elected to be members of the Executive Committee.

---

Dr. William Pepper, of Philadelphia, President of the Pan-American Medical Congress, addressed the American Medical Association at Detroit, on Thursday, June 9, 1892, upon the important mission of the Congress and the relations of the profession throughout the United States thereto. He spoke as follows:

Mr. President and Members of the American Medical Profession:

It is fitting I should appear thus before you, since it is to the authority of the American Medical Association that the Pan-American Medical Congress owes its existence; and since it is in the name of the entire American medical profession, that it proposes to secure the fullest recognition by the National Government.

From the moment that the suggestion of this Congress, to be held in the year 1893, ever memorable as the anniversary of Columbus's immortal discovery, was made by Dr. Reed, of Cincinnati, and his far-sighted and enthusiastic associates, it seemed to me a proposal of large importance. The hand of destiny is manifestly shaping the relations of all the countries of our Western Hemisphere, and no one can fail to recognize the close and cordial reciprocity which is to bind them together.

In no direction are such close reciprocal relations more desirable than between the medical professions of these countries. United as we are all, happily, coming to be in the maintenance of a standard of medical education which will soon challenge comparison with that of Europe, it is of practical moment that Canada, the United States, and the Latin-American countries shall grow familiar with the educational methods and facilities of each other. Possessing, as this vast stretch of territory does, the most admirable health resorts in every latitude, at all elevations, and adapted to every demand of medical science, it is important that opportunities shall be created for diffusing throughout the profession of the Hemisphere the fullest knowledge of these advantages.

United as these countries are destined to be in the most extensive commercial operations, there are problems of international hygiene and sanitation whose scientific discussion and wise solution will favorably affect the prosperity and happiness of great communities.

On behalf of my colleagues, who have honored me with a call to the important duty of presiding over the first Pan-American Medical Congress, I wish to assure you that we shall so administer the trust reposed in us that the large interests above mentioned shall be promoted.

The organization already effected seems so fully representative of the entire country, the indications of sympathy and favor on the part of the National Government are so encouraging, the responses already received from our neighboring countries are so numerous and cordial, that we may look forward with assured confidence to a great congress of the profession of the entire Western Hemisphere, united in fraternal spirit to promote these sacred purposes,—the advancement of science, the elevation of education, and the prevention and mitigation of disease.

His address was well received, and will do much to stimulate an interest in the Congress amongst the American medical profession.

Dr. Charles A. L. Reed, of Cincinnati, then offered the following, which was unanimously adopted :

*Resolved,* That the thanks of the American medical profession be and are hereby extended to the Hon. John Sherman, United States Senator for Ohio, and to the Senate Committee on Foreign Relations, for their services in securing the adoption of the Senate Bill (No. 76), authorizing and requesting the President to extend an invitation to the governments of the Western Hemisphere to send delegates to the Pan-American Medical Congress, and that concurrence in this resolution is hereby invoked at the hands of the House of Representatives and of the President of the United States.

---

WE HAVE spoken of the importance of good roads, in the columns of this journal on several occasions. The subject has been agitated all over the State by prominent citizens, but the interest has now become general throughout the country, and a memorial to Congress on the subject of a comprehensive exhibit of roads, their construction and maintenance, at the World's Columbian Exhibition, has been prepared under the direction of Col. Albert A. Pope, of Boston. This memorial is a brochure of 110 pages, consisting of sayings of prominent men on the subject of good roads, and paragraphs from newspapers and periodicals also relating to that subject. It is to be hoped that some action will be taken by the authorities in this State that will insure to its citizens a more substantial and better system of highways. The Empire State is noted for progress in all directions, but it is singularly backward in regard to its roads. Nothing could be more shockingly disgraceful than the country roads in most portions of the State during the Spring and Fall seasons. Physicians are greatly interested in this subject, hence we once more call attention to it.

---

Ex-Governor J. B. McCreary, representative in Congress from Kentucky, reported to the House on Thursday, July 7, 1892, a bill to authorize the President to invite the Government of Mexico and the Governments of the republics of Central and South America to send delegations, composed of prominent physicians, to the Pan-American Medical Congress, to be held in the City of Washington, in September, 1893. On Thursday, July 14th, Gov. McCreary asked unanimous consent for consideration of the bill, which was granted. After explaining its provisions, the bill was passed without a dissenting vote. Physicians throughout the whole country have taken great interest in this Congress, and especially in the passage of this bill, which gives it governmental sanction. The Pan-American Medical Congress, under the able officers chosen, seems to be moving to a surely successful issue. Physicians in Buffalo and vicinity who desire to become members should forward their initiation fee, $10, promptly to the Treasurer-General, Dr. A. M. Owen, of Evansville.

---

The Buffalo International Exposition will be held during the ten days beginning August 17th, and ending August 27, 1892. The fair consists of four departments, namely: A Southern Department, Department of Flowers and Plants, Department of Electrical Appliances, and a Department pertaining to Live Stock. The admission fee at the gate will be fifty cents, and the manager, Mr.

Geo. M. Robinson, who is experienced in the conduct of expositions, is entitled to encouragement in this Buffalo enterprise. We acknowledge an invitation to an old-time Southern possum supper, to be given on the grounds of the exposition in the afternoon of August 17th, in the Southern department of which the Hon. John D. Patrick, of Raleigh, N. C., is manager.

---

The Second Bulletin of the Harvard Medical School Association, together with the Catalogue of the Alumni, has appeared. These bulletins are the handsomest alumni records that we have seen, and reflect credit upon the famous institution and its distinguished alumni association. The bulletins may be obtained upon application to the secretary, Dr. Robert Williamson Lovett, 379 Boylston street, Boston, Mass.

